# Artificial intelligence-based joint attenuation and scatter correction strategies for multi-tracer total-body PET

**DOI:** 10.1186/s40658-024-00666-8

**Published:** 2024-07-19

**Authors:** Hao Sun, Yanchao Huang, Debin Hu, Xiaotong Hong, Yazdan Salimi, Wenbing Lv, Hongwen Chen, Habib Zaidi, Hubing Wu, Lijun Lu

**Affiliations:** 1 School of Biomedical Engineering, Southern Medical University, 1023 Shatai Road, Guangzhou, 510515 China; 2 Division of Nuclear Medicine and Molecular Imaging, Geneva University Hospital, 1211 Geneva 4, Switzerland; 3 Laboratory for Quality Control and Evaluation of Radiopharmaceuticals, Department of Nuclear Medicine, Nanfang Hospital Southern Medical University, Guangzhou, 510515 China; 4 Department of Medical Engineering, Nanfang Hospital Southern Medical University, Guangzhou, 510515 China; 5 Guangdong Provincial Key Laboratory of Medical Image Processing, Southern Medical University, 1023 Shatai Road, Guangzhou, 510515 China; 6 Guangdong Province Engineering Laboratory for Medical Imaging and Diagnostic Technology, Southern Medical University, 1023 Shatai Road, Guangzhou, 510515 China; 7 Department of Electronic Engineering, Information School, Yunnan University, Kunming, 650091 China; 8 Pazhou Lab, Guangzhou, 510330 China

**Keywords:** Artificial intelligence, PET/CT, Total-body, Attenuation correction, Scatter correction

## Abstract

**Background:**

Low-dose ungated CT is commonly used for total-body PET attenuation and scatter correction (ASC). However, CT-based ASC (CT-ASC) is limited by radiation dose risks of CT examinations, propagation of CT-based artifacts and potential mismatches between PET and CT. We demonstrate the feasibility of direct ASC for multi-tracer total-body PET in the image domain.

**Methods:**

Clinical uEXPLORER total-body PET/CT datasets of [^18^F]FDG (*N* = 52), [^18^F]FAPI (*N* = 46) and [^68^Ga]FAPI (*N* = 60) were retrospectively enrolled in this study. We developed an improved 3D conditional generative adversarial network (cGAN) to directly estimate attenuation and scatter-corrected PET images from non-attenuation and scatter-corrected (NASC) PET images. The feasibility of the proposed 3D cGAN-based ASC was validated using four training strategies: (1) Paired 3D NASC and CT-ASC PET images from three tracers were pooled into one centralized server (CZ-ASC). (2) Paired 3D NASC and CT-ASC PET images from each tracer were individually used (DL-ASC). (3) Paired NASC and CT-ASC PET images from one tracer ([^18^F]FDG) were used to train the networks, while the other two tracers were used for testing without fine-tuning (NFT-ASC). (4) The pre-trained networks of (3) were fine-tuned with two other tracers individually (FT-ASC). We trained all networks in fivefold cross-validation. The performance of all ASC methods was evaluated by qualitative and quantitative metrics using CT-ASC as the reference.

**Results:**

CZ-ASC, DL-ASC and FT-ASC showed comparable visual quality with CT-ASC for all tracers. CZ-ASC and DL-ASC resulted in a normalized mean absolute error (NMAE) of 8.51 ± 7.32% versus 7.36 ± 6.77% (*p* < 0.05), outperforming NASC (*p* < 0.0001) in [^18^F]FDG dataset. CZ-ASC, FT-ASC and DL-ASC led to NMAE of 6.44 ± 7.02%, 6.55 ± 5.89%, and 7.25 ± 6.33% in [^18^F]FAPI dataset, and NMAE of 5.53 ± 3.99%, 5.60 ± 4.02%, and 5.68 ± 4.12% in [^68^Ga]FAPI dataset, respectively. CZ-ASC, FT-ASC and DL-ASC were superior to NASC (*p* < 0.0001) and NFT-ASC (*p* < 0.0001) in terms of NMAE results.

**Conclusions:**

CZ-ASC, DL-ASC and FT-ASC demonstrated the feasibility of providing accurate and robust ASC for multi-tracer total-body PET, thereby reducing the radiation hazards to patients from redundant CT examinations. CZ-ASC and FT-ASC could outperform DL-ASC for cross-tracer total-body PET AC.

## Background

Whole-body PET scanning using [^18^F]-fluorodeoxyglucose ([^18^F]FDG) is commonly used for diagnosis, staging, restaging and monitoring of response to treatment in clinical oncology [1]. [^18^F]-fibroblast-activation protein inhibitors ([^18^F]FAPI) and [^68^Ga]-fibroblast-activation protein inhibitors ([^68^Ga]FAPI) PET are recently performed in clinical settings, showing great potential for widespread oncologic application [2]. Quantitative and semi-quantitative metrics in PET, such as the standardized uptake value (SUV), play an important role in providing valuable information for disease diagnosis and therapy monitoring in the field of oncology [3]. Accurate corrections for physical degrading factors, such as attenuation and Compton scattering, are essential for reliable quantitative PET imaging [4].

Total-body PET/CT scanners have been used in clinical practice, showing great potential for low-dose imaging, faster scanning and whole-body dynamic imaging [5]. On commercial hybrid total-body PET/CT scanners, the CT component can be used for PET image attenuation and scatter correction (ASC), anatomical localization and clinical diagnosis [5]. Although routine PET/CT scans follow the “As Low As Reasonably Achievable” (ALARA) principle [6], the risk of ionizing radiation from CT remains a matter of concern, and even routine low-dose CT has been reported to contribute 6.4 mSv [7]. This problem of radiation dose is further accentuated by the increased long axial field of view (LAFOV) in total-body PET/CT scanners [8]. A nationwide survey in South Korea reported a mean effective dose from the CT component of 6.26 ± 3.06 mSv of various diagnostic PET/CT procedures [9]. Another study reviewed PET/CT scans of 210 patients and found that CT contributed to 69% of the total effective dose [10]. Therefore, the issue of radiation dose from CT needs to be considered in PET/CT scanning.

Ultra-low-dose CT attenuation correction (AC) has shown great potential to significantly reduce radiation exposure in whole-body (2.1 mSv) [11] and total-body (reducing radiation dose by more than 90%) [12] PET/CT scanning. Although low-dose CT scans are widely used for PET ASC, CT-less PET ASC remains essential in many situations. In the case of ultra-low-dose PET scans, CT radiation dose becomes a limiting factor restricting the low dose capability of total-body PET imaging [12]. Additionally, patients undergoing multi-tracer PET examinations face increased radiation safety risks [13], especially for subjects requiring multiple time points imaging, such as [^89^Zr]-based antibody tracer studies [14–16]. Pregnant women and pediatric patients, who are more radiation-sensitive, would benefit from CT-less PET scanning [17]. Furthermore, CT-based ASC (CT-ASC) is limited by the propagation of CT-based artifacts and potential mismatch between PET and CT [18]. CT-ASC requires an additional PET image reconstruction step, which imposes a greater demand for computational resources and increases the reconstruction time for total-body PET/CT in routine clinical practice. Therefore, CT-less ASC methods for total-body PET would be of great benefit in the clinic.

Several CT-less ASC methods have been developed for PET/MR scanners since MRI cannot directly provide the photon attenuation information needed for PET ASC, including segmentation-based [19] and atlas-based [20] techniques. Nevertheless, these strategies are limited by tissue misclassification, intra/inter-atlas misregistration and anatomic abnormalities. Nutys et al. [21] proposed a maximum-likelihood reconstruction of attenuation and activity (MLAA) method to simultaneously reconstruct tracer activity and attenuation maps (µ-maps) without relying on CT or MRI structural information. However, even with the introduction of time-of-flight (TOF) information, MLAA is still limited by high noise and the insufficient coincidence time resolution of current clinical PET systems [22]. The MLAA-based AC method also faces the limitation of the chicken-egg dilemma in scatter estimation [23]. Cheng et al. [24] proposed a new maximum likelihood activity and attenuation reconstruction method that utilizes both TOF PET data and transmission data from lutetium-176 background radiation (MLAA-TX), which outperformed the standard MLAA reconstruction. The feasibility of joint reconstruction algorithms using lutetium background for AC has been studied in LAFOV PET scanners, including Siemens Biograph Vision Quadra scanner [25] and uEXPLORER total-body PET scanner [26].

In recent years, artificial intelligence (AI) has shown promising potential to address the limitations of conventional ASC techniques in PET [27]. Multiple studies explored the feasibility of generating pseudo-CTs or μ-maps from MR images [28–31] for PET ASC in the brain and pelvic regions. Several deep learning (DL) approaches were developed to generate pseudo-CT [32–34] or ASC PET images [35–38] from non-attenuation-corrected (NAC) PET images for brain or whole-body PET. Other DL approaches were developed to improve the quality of the MLAA μ-maps and the corresponding activity image [39–44]. However, these DL methods primarily focus on specific tracers and may be limited in their robustness to new tracers due to constraints in the size of training datasets. The rapid advancement of novel tracers in PET imaging presents challenges in efficiently obtaining substantial clinical data to train network models, thereby impeding the robustness and reliability of DL-based AC methods. Toyonaga et al. [42] proposed a 3D U-net framework for multi‑tracer whole‑body PET AC. Hwang et al. [43] compared two DL-based AC approaches using two tracers in whole-body PET. These two studies trained individual networks for each tracer, while the application of these individual networks in cross-tracer PET AC was not investigated. Hashimoto et al. [45] found that a convolutional neural network (CNN) trained on a mixed dataset of six radiotracers outperformed CNNs trained on split datasets generated from each individual radiotracer for brain PET AC. Guo et al. [46] proposed integrating domain knowledge in DL for CT-free PET imaging, achieving efficient and robust performance of ASC on cross-scanner or cross-tracer PET images. The robustness of DL-based AC for multi-tracer applications on total-body PET scanners requires further validation, as the attenuation correction factors (ACFs) can exceed 100 or more in scanners with LAFOV [47]. Fine-tuning (FT) strategy has been reported to improve the clinical adaption of DL-based AC on new scanners and tracers for myocardial perfusion (MP) SPECT [48]. Our previous work [49] also demonstrated that FT showed promising potential for dynamic MP PET. There are no reports on the application of FT for total-body PET ASC.

In this study, we demonstrated the feasibility of robust CT-less ASC for multi-tracer total-body PET using different AI-based ASC strategies. We developed an improved 3D conditional generative adversarial network (cGAN) to generate attenuation and scatter-corrected PET images directly from non-attenuation and scatter-corrected (NASC) PET images. The proposed methods can reduce the radiation risk to patients from redundant CT examinations. We aim to propose this development for potential applications on CT-less total-body PET scanners and enhance the accuracy and reliability of such scanners. We are committed to exploring AI-based ASC strategies that are generalizable across different tracers and clinical scenarios.

## Methods

### Patient characteristics and image acquisition

This study retrospectively recruited 158 subjects who underwent total-body PET/CT examinations on a uEXPLORER total-body PET/CT scanner (United Imaging Healthcare, China) at the Nanfang PET Center, Nanfang Hospital, including [^18^F]FDG (*N* = 52), [^18^F]FAPI (*N* = 46) and [^68^Ga]FAPI (*N* = 60) studies. We thoroughly inspected all datasets before inclusion, excluding instances with obvious artifacts, poor image quality, or missing CT and/or NASC images. This study was performed in line with the principles of the Declaration of Helsinki. The study was approved by the local institutional review board, and the need for written informed consent was waived. For each patient, a low-dose CT scan was performed before the total-body PET scan and converted to the attenuation map using a bilinear model [50]. A 5-min total-body PET examination was then performed for the patient. Scatter correction was performed only on CT-ASC PET images using the Monte Carlo-based algorithm [51]. The PET images were reconstructed using the ordered subset expectation maximization (OSEM) algorithm with 3 iterations and 20 subsets, incorporating TOF and point-spread function (PSF) modeling on a medical image processing workstation (uWS-MI, United Imaging Healthcare). The deadtime, normalization and decay corrections were also performed. The attenuation map was registered to the corresponding PET data with no observed mismatches. Detailed patient demographics, image acquisition and reconstruction settings can be found in Table 1.

**Table 1:** Patient demographics, total-body PET/CT image acquisition and reconstruction settings

Scanner	United imaging healthcare uEXPLORER		
Tracer	[^18^F]FDG	[^18^F]FAPI	[^68^Ga]FAPI
Patient number	52	46	60
Age (year)	55.4 ± 16.9 (17–81)	55.8 ± 14.5 (17–83)	52.2 ± 12.2 (23–75)
Gender	34 male/18 female	36 male/10 female	31 male/29 female
BMI	22.89 ± 3.65	22.04 ± 3.48	21.38 ± 3.38
Injection activity (MBq)	232.73 ± 45.74	144.90 ± 29.91	80.08 ± 30.49
Scanning time (s)	300		
Scatter correction	Monte Carlo simulation		
Reconstruction	OSEM, 3 iterations × 20 subsets		
PET matrix/voxel size (mm^3^)	192 × 192 × 673/3.125 × 3.125 × 2.886		
CT scan matrix/voxel size (mm^3^)	512 × 512 × 673/1.367 × 1.367 × 3 (mm^3^)		
CT scan	120 kVp, dynamic (179.6 ± 81.7) mAs, rotation time 0.5 s, pitch 1.0125	120 kVp, 80 mAs, rotation time 0.5 s, pitch 1.0125	100 kVp, 80 mAs, rotation time 0.5 s, pitch 1.0125

### Image preprocessing

In our implementation, the voxel values of all NASC and CT-ASC total-body PET images were converted to SUV to reduce the dynamic range of the intensity of PET images, which can facilitate effective training of the models [52]. Subsequently, all 3D NASC and CT-ASC total-body PET images were cropped to a fixed patch size of 192 × 192 × 64 across axial slices with a sliding window of 32-slice overlap. After the testing steps, the network output would be stitched to obtain the complete total-body PET image, and the overlapping regions would be averaged to mitigate boundary artifacts resulting from patch concatenation. Four kinds of data augmentation were performed for all training data using the Augmentor3D package (https://github.com/amogh3892/Augmentor3D), including rotation with 10°, horizontal flipping, translation with (5, 5, 0) voxels and shearing with (0.05, 0.05) magnitude.

### Network architectures

**Fig. 1:**
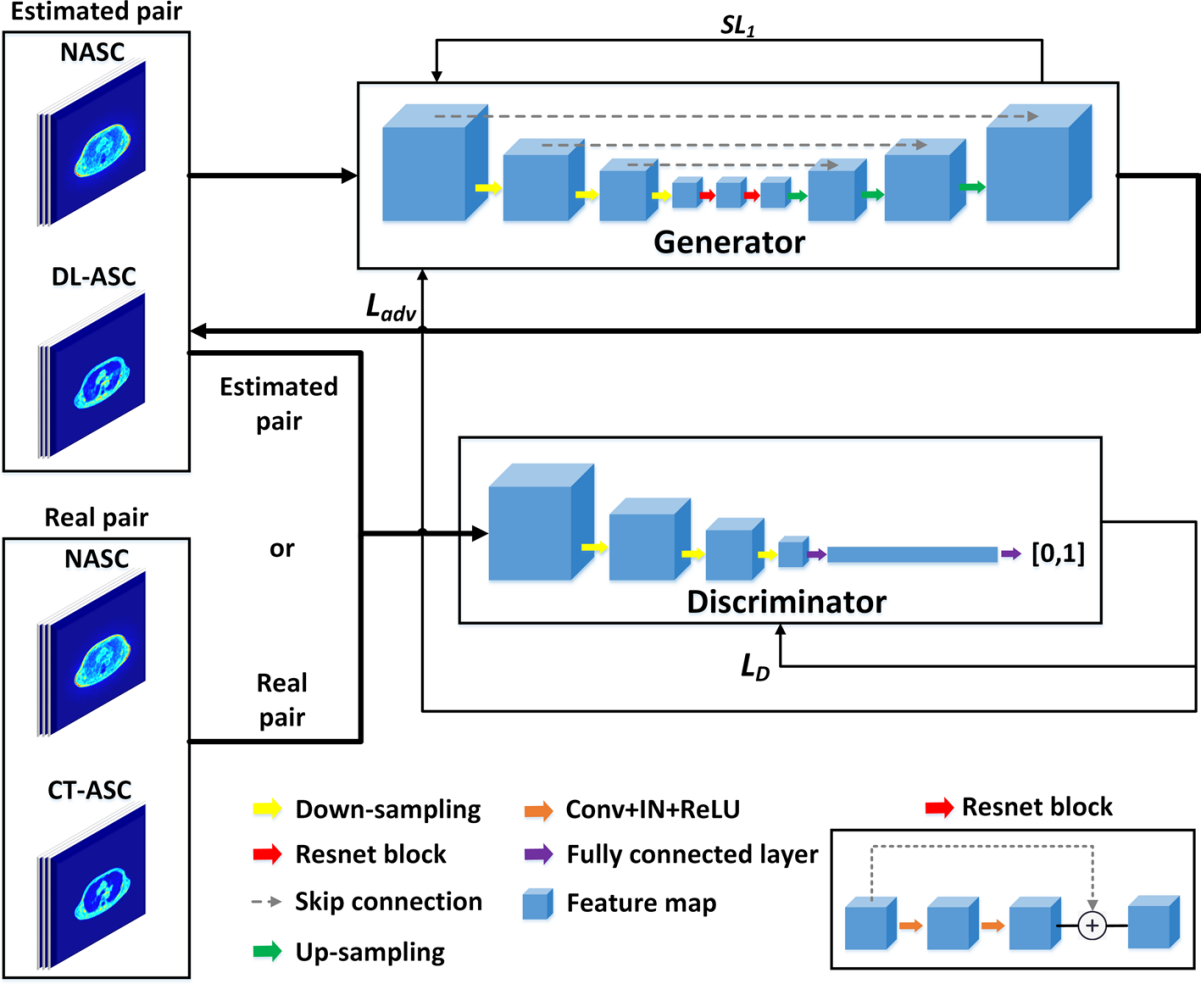
Schematic diagram of the 3D cGAN architecture

We implement an improved 3D cGAN comprising a discriminator D and a generator G, as depicted in Fig. 1. The generator loss *L*_*G*_ and the discriminator loss *L*_*D*_ are defined as follows:

1 $$L_{G} (x,y) = L_{adv} (x) + \lambda SL_{1} (G(x),y)$$

2 $$L_{D} (x,y) = \frac{1}{2}\left( {\left( {D(x,y) - T_{real} } \right)^{2} + \left( {D(x,G(x)) - T_{synthetic} } \right)^{2} } \right)$$

where *x* is the NASC PET image, *y* is the target CT-ASC PET image. *L*_*adv*_ is the adversarial loss function of the generator. *SL*_1_ is the smooth *L*_1_ loss function, which converges rapidly and is insensitive to outliers [53]. *L*_*adv*_ and *SL*_1_ are defined as:

3 $$L_{adv} (x) = \frac{1}{2}\left( {D(x,G(x)) - T_{real} } \right)^{2}$$

4 $$SL_{1} (x,y) = \left\{ {\begin{array}{*{20}l} {0.5(y - G(x))^{2} ,} \hfill & {\left| {y - G(x)} \right| < 1} \hfill \\ {\left| {y - G(x)} \right| - 0.5,} \hfill & {otherwise} \hfill \\ \end{array} } \right.$$

where *T*_*real*_ = 1 and *T*_*synthetic*_ = 0 are labels for the discriminant results of real and synthetic images, respectively. *λ* is the weight for *SL*_1_ loss and is set to 10 in this study.

The generator G consisted of a 3D U-net with three encoder-decoder layers and a ResNet with two blocks. The encoder and decoder consisted of a series of convolutional layers with 3 × 3 × 3 kernels, followed by an instance normalization (IN) layer and a rectified linear unit (ReLU) activation function. A convolution layer with a stride of 2 and 3 × 3 × 3 kernels was used for down-sampling. The number of feature channels was doubled in each down-sampling step. A bilinear interpolation was used for each up-sampling step, followed by a convolutional layer with and 3 × 3 × 3 kernels, and the number of feature channels was halved. Skip connections were used where the output of 1st and 3rd layers in the encoder was concatenated with the corresponding layer in the decoder. After two down-sampling steps with a 0.5 dropout ratio, the residual blocks were extracted to extract the deep features. The discriminator was a convolutional neural network (CNN) architecture consisting of 4 3 × 3 × 3 convolutional layers, a fully connected layer and a sigmoid layer. The first convolution layer of the discriminator consisted of 64 3 × 3 × 3 kernels convolutions with stride 2, followed by the leaky rectified linear unit (LReLU) function. The 2nd to 4th convolutional layers were followed by a batch normalization (BN) layer and the LReLU function. The slope of the LReLU function is 0.2. The number of convolution kernels in the following layers was twice of the previous convolution layers. We implemented the 3D cGAN using Pytorch on a Linux workstation with an NVIDIA RTX 4090 GPU (24 GB). The Adam optimizer was applied for both the generator and discriminator.

### Network training

**Fig. 2:**
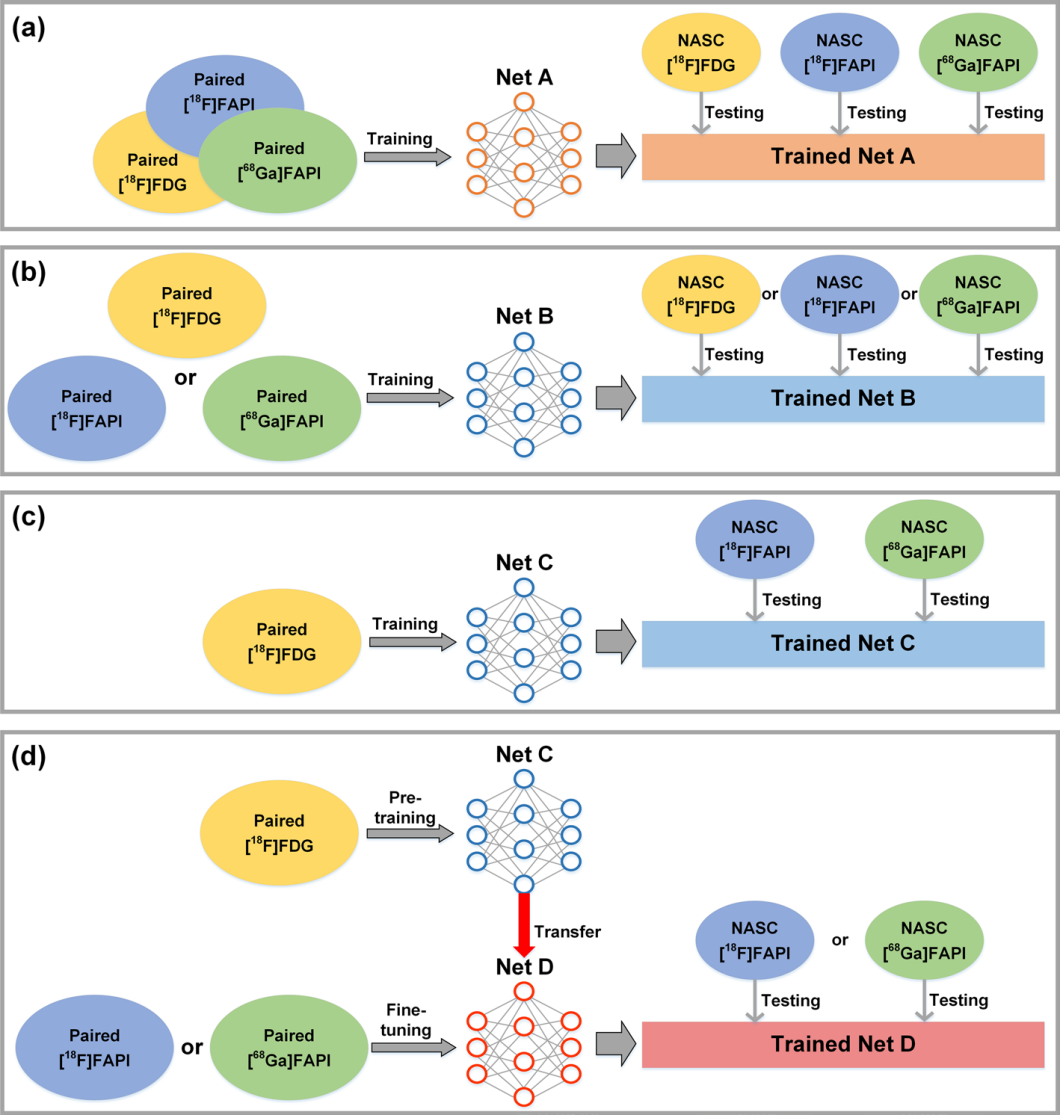
Schematic diagrams of **a** CZ-ASC, **b** DL-ASC, **c** NFT-ASC and **d** FT-ASC methods used in this study

We trained all networks in fivefold cross-validation. In each fold, the number of data in the training, validation and testing dataset was 35 (4 augmentation methods + original):1:2. Paired 3D NASC and CT-ASC PET images were used as the network input and label, respectively. Four network training strategies were performed and compared: (1) The 3D cGAN was trained on all three tracer datasets and tested with all three tracers (CZ-ASC); (2) The 3D cGAN was trained on each tracer dataset individually and tested with the same tracer (DL-ASC); (3) The 3D cGAN was trained with paired [^18^F]FDG data only and tested with [^18^F]FAPI and [^68^Ga]FAPI datasets (NFT-ASC); (4) The 3D cGAN is pre-trained with paired [^18^F]FDG data only and then fine-tuned with one of the [^18^F]FAPI and [^68^Ga]FAPI datasets and tested with the same FAPI tracer (FT-ASC). Figure 2 shows the schematic diagrams of CZ-ASC, DL-ASC, NFT-ASC, and FT-ASC. All network models were trained for 300 epochs with a mini-batch of 2 images. An adaptive learning rate was used for all ASC methods, which started with an initial value of 0.0001 and employed linear decay as the epochs increased. The outputs of each ASC network model were merged to generate the full total-body PET data for all subjects.

### Evaluation metrics

We evaluated different ASC methods by qualitative and quantitative assessments using CT-ASC total-body PET as the reference. For voxel-based analysis, normalized mean absolute error (NMAE), normalized mean square error (NMSE), peak signal-to-noise ratio (PSNR), and structural similarity index (SSIM) were quantified on different ASC methods using CT-ASC as the reference:

5 $$NMAE = {{\sum\limits_{i = 1}^{N} {\left| {x(i) - y(i)} \right|} } \mathord{\left/ {\vphantom {{\sum\limits_{i = 1}^{N} {\left| {x(i) - y(i)} \right|} } {\sum\limits_{i = 1}^{N} {\left| {y(i)} \right|} }}} \right. \kern-0pt} {\sum\limits_{i = 1}^{N} {\left| {y(i)} \right|} }}$$

6 $$NMAE = {{\sum\limits_{i = 1}^{N} {\left( {x(i) - y(i)} \right)^{2} } } \mathord{\left/ {\vphantom {{\sum\limits_{i = 1}^{N} {\left( {x(i) - y(i)} \right)^{2} } } {\sum\limits_{i = 1}^{N} {\left( {y(i)} \right)^{2} } }}} \right. \kern-0pt} {\sum\limits_{i = 1}^{N} {\left( {y(i)} \right)^{2} } }}$$

7 $$PSNR = 10 \cdot \log_{10} \left( {\frac{{MAX_{y}^{2} }}{MSE(x,y)}} \right)$$

8 $$SSIM = \frac{{(2\mu_{x} \mu_{y} + C_{1} )(2\sigma_{xy} + C_{2} )}}{{\left( {\mu_{x}^{2} + \mu_{y}^{2} + C_{1} } \right)\left( {\sigma_{x}^{2} + \sigma_{y}^{2} + C_{2} } \right)}}$$

where *x* indicates the predicted image, *y* indicates the reference image, *N* indicates the total number of voxels, whereas *i* is the voxel index. *MAX*_*y*_ is the maximum voxel value of the reference image while *MSE*(*x*, *y*) is the mean squared error between predicted and reference images. *μ*_*x*_ and *μ*_*y*_ denote the mean value of the predicted image and the reference image. $\sigma_{x}^{2}$ and $\sigma_{y}^{2}$ are the variances of the predicted image and the reference image, whereas $\sigma_{xy}$ indicates their covariance. The parameters *C*_1_ = (*k*_1_*I*)^2^ and *C*_2_ = (*k*_2_*I*)^2^ with constants *k*_1_ = 0.01 and *k*_2_ = 0.03 were used in this work, and *I* represents the maximum intensity of the reference image.

We also investigated the robustness of different methods to the in vivo uptake variation for all patients. Inspired by [38, 42], in addition to complete total-body PET images, we evaluated within five sub-regions: head and neck, chest, abdomen, pelvis, and leg, which correspond to 0–20%, 20–40%, 40–55%, 55–70%, and 70–100% of the image volume, respectively. A paired *t*-test with Bonferroni correction was used for statistical analysis to evaluate the NMAE, NMSE, PSNR, and SSIM results in full total-body PET images for different ASC methods. A *p*-value < 0.05 indicates a significant difference. Furthermore, joint correlation histogram and linear regression were evaluated for NASC and different ASC methods.

In this study, we use a multiple-organ segmentation algorithm [54] to segment a total of 23 different organs from total-body CT images for each patient in the three datasets. The CT masks were then resampled to match the corresponding PET images. Considering the imaging characteristics of different tracers, we evaluated the brain, kidney, liver, lung, bladder, and whole heart regions for each patient in the [^18^F]FDG dataset. Similarly, we assessed the kidney, liver, lung, bladder, and whole heart regions for each patient in the [^18^F]FAPI and [^68^Ga]FAPI datasets. Using CT-ASC as a reference, we evaluated the absolute percentage differences in SUV_max_ (SUV_max__diff) and SUV_mean_ (SUV_mean__diff) for CZ-ASC, DL-ASC, NFT-ASC, and FT-ASC methods across different regions. The SUV_max__diff and SUV_mean__diff are defined as:

9 $$SUV_{\max } \_diff = \frac{{\left| {SUV_{\max } \_predicted - SUV_{\max } \_ref} \right|}}{{\left| {SUV_{\max } \_ref} \right|}} \times 100\%$$

10 $$SUV_{mean} \_diff = \frac{{\left| {SUV_{mean} \_predicted - SUV_{mean} \_ref} \right|}}{{\left| {SUV_{mean} \_ref} \right|}} \times 100\%$$

where *SUV*_*max*_*_predicted* and *SUV*_*mean*_*_predicted* are the SUV_max_ and SUV_mean_ of a region of interest (ROI) in the predicted image, respectively. *SUV*_*max*_*_ref* and *SUV*_*mean*_*_ref* are the SUV_max_ and SUV_mean_ of a ROI of CT-ASC, respectively. A paired *t*-test with Bonferroni correction was used for statistical analysis to evaluate the SUV_max__diff and SUV_mean__diff results in the target regions between CZ-ASC and other methods. A *p*-value < 0.05 indicates a significant difference.

## Results

**Fig. 3:**
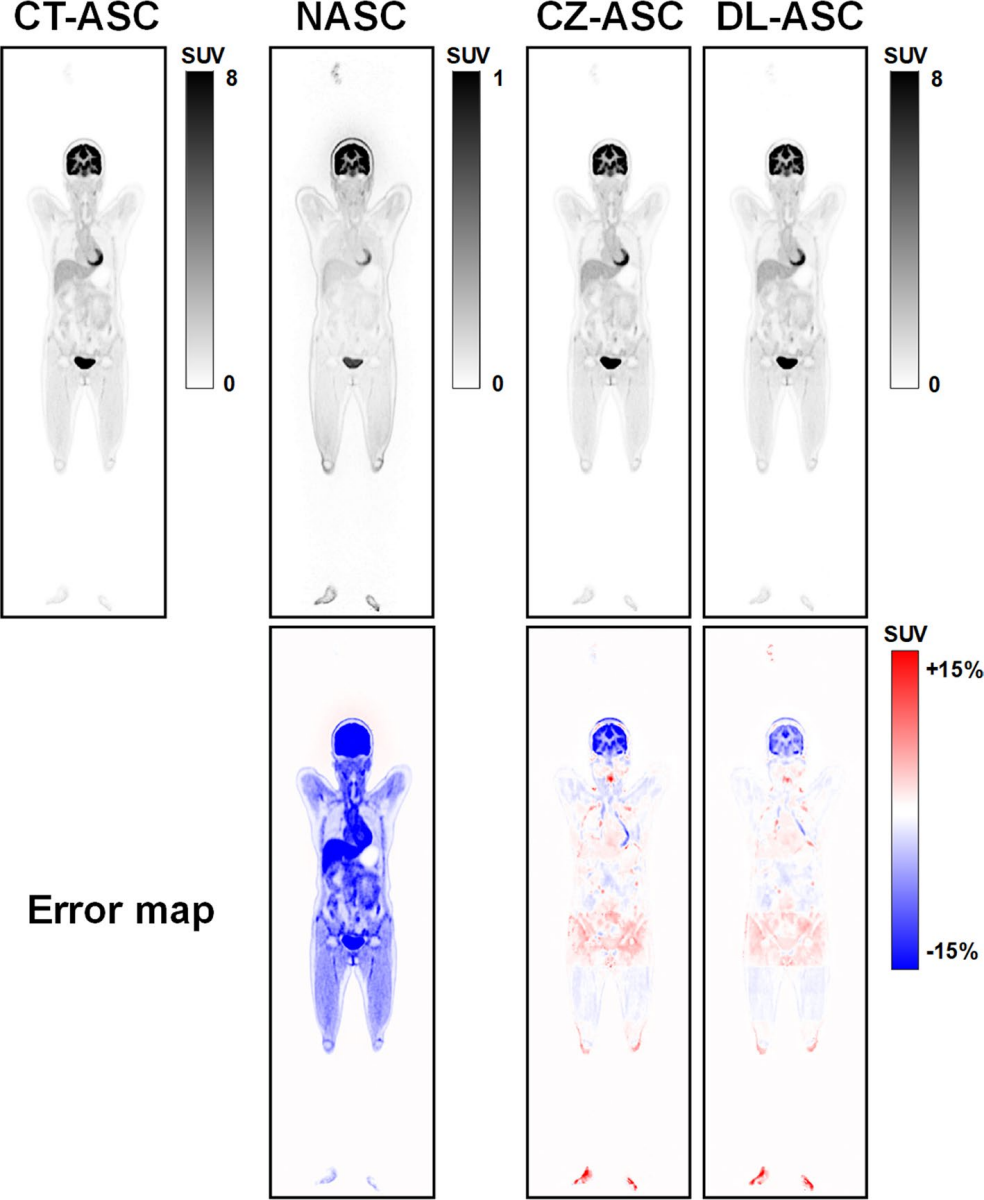
Sample coronal total-body PET images of CT-ASC, NASC, CZ-ASC and DL-ASC methods for a female patient (Age: 51, BMI: 22.72) in the [^18^F]FDG dataset

Figure 3 shows sample coronal slices of CT-ASC, NASC, CZ-ASC and DL-ASC of a woman from the [^18^F]FDG dataset. The corresponding error maps are also shown in terms of relative percentage error range [− 15%, 15%]. CZ-ASC shows relatively large errors in the brain and pelvis regions, while DL-ASC shows smaller errors compared to CZ-ASC in these regions. Both CZ-ASC and DL-ASC outperform NASC.

**Fig. 4:**
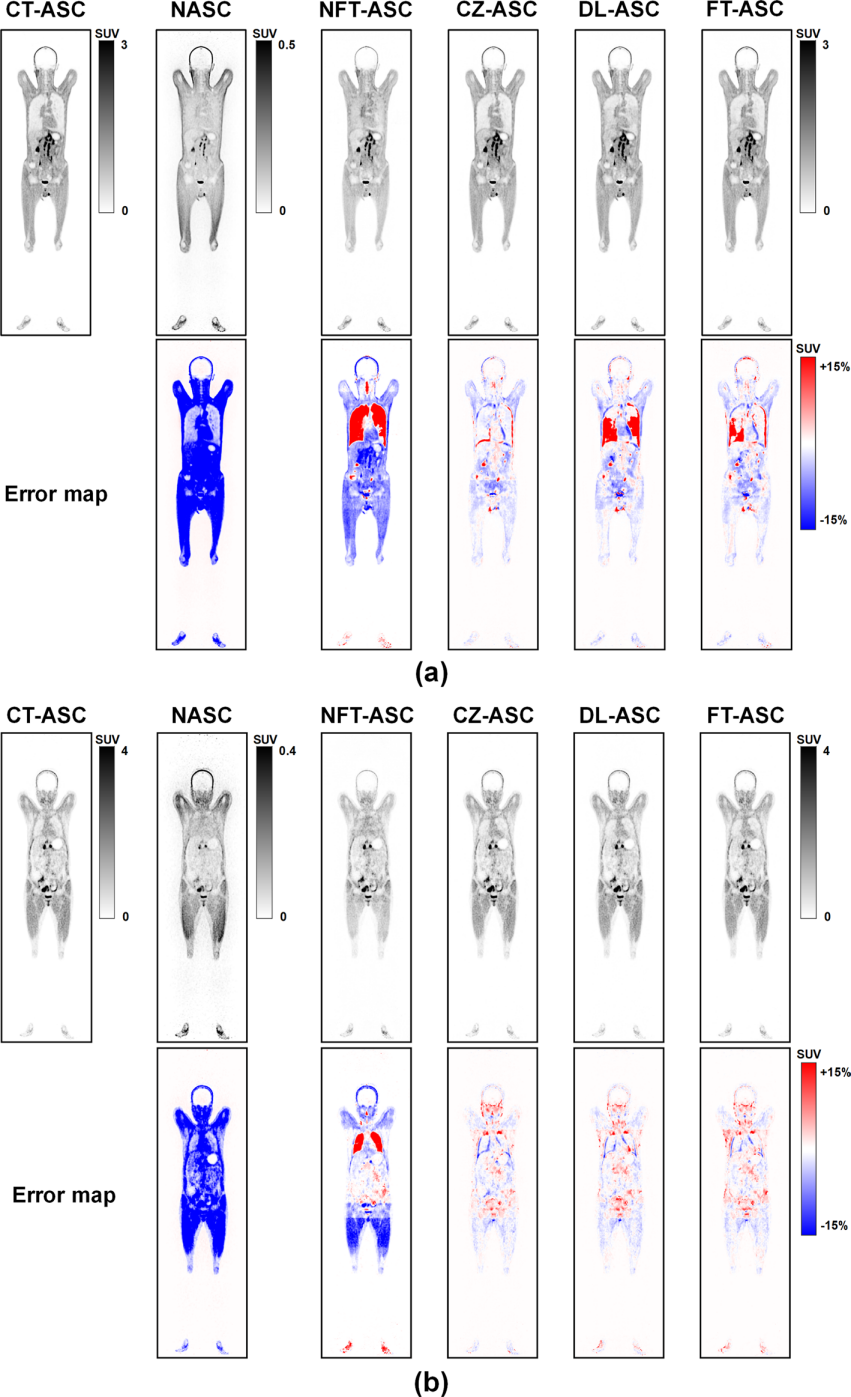
Sample coronal total-body PET images of CT-ASC, NASC, NFT-ASC, CZ-ASC, DL-ASC and FT-ASC methods for **a** a male patient (Age: 41, BMI: 21.41) in the [^18^F]FAPI and **b** a female patient (Age: 52, BMI: 21.72) in the [^68^Ga]FAPI dataset

Figure 4 illustrates the sample coronal results of CT-ASC, NASC, NFT-ASC, CZ-ASC, DL-ASC and FT-ASC methods for a man and a woman in the [^18^F]FAPI and [^68^Ga]FAPI dataset, respectively. The corresponding error maps are all estimated using CT-ASC as the reference and shown in terms of relative percentage error range [− 15%, 15%]. CZ-ASC, DL-ASC, and FT-ASC methods improve tumor detection performance for a man with extensive lymph node metastases and a woman with gastric and ovarian malignancies as compared to NASC. These two cases of metastases showed the adaptability of CZ-ASC, DL-ASC and FT-ASC methods for various lesion locations and sizes throughout the body. CZ-ASC shows the best performance for the two subjects. DL-ASC shows relatively large errors in the pulmonary region, while FT-ASC shows smaller errors in this region compared to DL-ASC. NFT-ASC shows significant overestimation in the pulmonary region and significant underestimation in other regions, yet outperforming NASC.

**Fig. 5:**
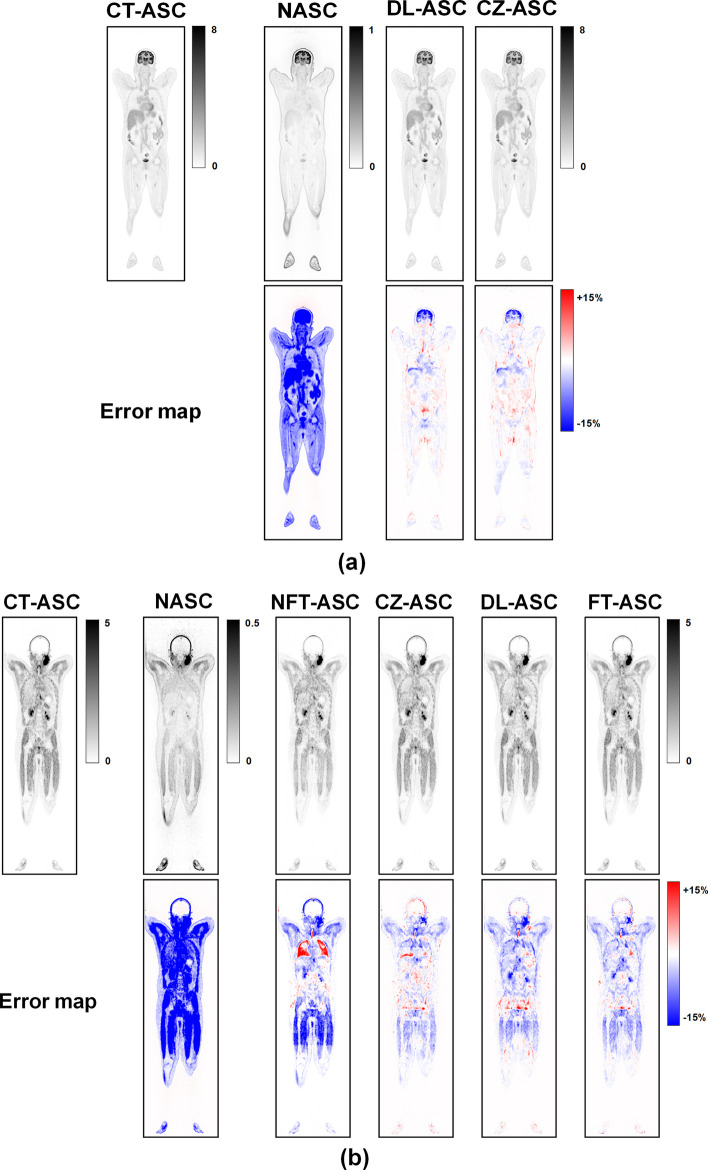
Sample results of different ASC methods for **a** an obese male patient (Age: 53, BMI: 30.85) in the [^18^F]FDG dataset and **b** an obese male patient (Age: 24, BMI: 33.22) in the [^68^Ga]FAPI dataset

Figure 5 shows the sample results of different ASC methods for two obese subjects. These two cases of obesity show that CZ-ASC, DL-ASC and FT-ASC have a robust ability to correct for subjects with a high BMI, which may have substantial attenuation and scatter caused by the longer photon penetration distance. CZ-ASC and DL-ASC show similar visual results yet outperform NASC for the man in the [^18^F]FDG dataset. For the man in the [^68^Ga]FAPI dataset, DL-ASC shows significant errors in the neck lymph nodes, kidneys, and bladder regions. FT-ASC shows smaller errors in these regions compared to DL-ASC. CZ-ASC performs the best overall. NFT-ASC shows significant errors in the head & neck, lungs, kidneys, and thighs, but it outperforms NASC.

**Fig. 6:**
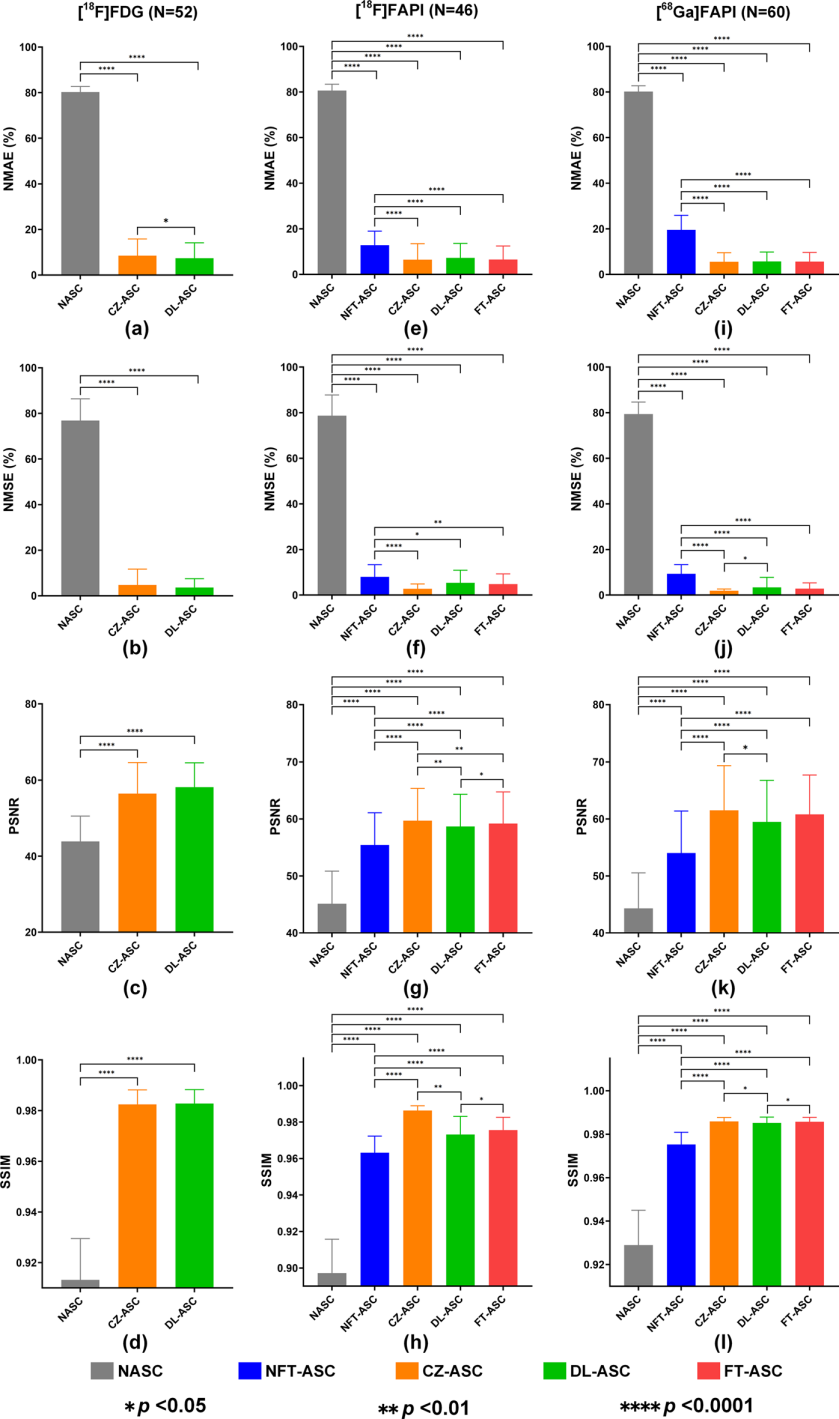
Results of paired *t*-test with Bonferroni correction for different ASC methods in the **a**–**d** [^18^F]FDG, **e**–**h** [^18^F]FAPI, and **i**–**l** [^68^Ga]FAPI datasets in terms of NMAE, NMSE, PSNR and SSIM. A *p*-value < 0.05 indicates a significant difference

Table 2 provides the detailed NMAE, NMSE, PSNR and SSIM results of different methods across all subjects in the three tested datasets, and they are consistent with visual image results. Figure 6 shows the paired *t*-test results for different ASC methods in terms of NMAE, NMSE, PSNR and SSIM. For the [^18^F]FDG dataset, CZ-ASC and DL-ASC are better than NASC in terms of all voxel-based metrics (*p* < 0.0001). CZ-ASC shows worse performance than DL-ASC but with no significant difference for the NMSE (CZ-ASC: 4.77 ± 6.91%, DL-ASC: 3.66 ± 3.83%), PSNR (CZ-ASC: 56.46 ± 8.17, DL-ASC: 58.18 ± 6.39) and SSIM (CZ-ASC: 0.9825 ± 0.0057, DL-ASC: 0.9828 ± 0.0055) results (*p* > 0.05). CZ-ASC, DL-ASC and FT-ASC are better than NASC and NFT-ASC in terms of all metrics for [^18^F]FAPI and [^68^Ga]FAPI datasets. CZ-ASC shows the best performance in [^18^F]FAPI and [^68^Ga]FAPI datasets, while FT-ASC outperforms DL-ASC in these two datasets. For the statistical *t*-test results, all voxel-based metrics of CZ-ASC, DL-ASC and FT-ASC are significantly better than NASC (*p* < 0.0001) and NFT-ASC (*p* < 0.05) for the two datasets. CZ-ASC outperforms DL-ASC significantly in the NMSE (CZ-ASC: 1.92 ± 0.76%, DL-ASC: 3.41 ± 4.38%), PSNR (CZ-ASC: 61.51 ± 7.82, DL-ASC: 59.46 ± 7.31) and SSIM (CZ-ASC: 0.9859 ± 0.0018, DL-ASC: 0.9852 ± 0.0027) results (*p* < 0.05) for the [^68^Ga]FAPI dataset, as well as in the PSNR (CZ-ASC: 59.68 ± 5.67, DL-ASC: 58.66 ± 5.66) and SSIM (CZ-ASC: 0.9863 ± 0.0026, DL-ASC: 0.9732 ± 0.0099) results (*p* < 0.01) for the [^18^F]FAPI dataset. FT-ASC outperforms DL-ASC significantly in the PSNR (FT-ASC: 59.18 ± 5.56) and SSIM (FT-ASC: 0.9756 ± 0.0070) results (*p* < 0.05) for the [^18^F]FAPI dataset and in the SSIM (FT-ASC: 0.9857 ± 0.0021) results (*p* < 0.05) for the [^68^Ga]FAPI dataset. No significant difference is observed in the NMAE ([^18^F]FAPI: CZ-ASC: 6.44 ± 7.02%, DL-ASC: 7.25 ± 6.33%, FT-ASC: 6.55 ± 5.89%; [^68^Ga]FAPI: CZ-ASC: 5.53 ± 3.99%, DL-ASC: 5.68 ± 4.12%, FT-ASC: 5.60 ± 4.02%) results (*p* > 0.05) among CZ-ASC, DL-ASC, and FT-ASC. NFT-ASC is better than NASC in terms of all voxel-based metrics (*p* < 0.0001).

**Table 2:** Comparison of NMAE, NMSE, PSNR and SSIM results (mean ± SD) of complete total-body PET images for different methods

Datasets	Methods	NMAE (%)	NMSE (%)	PSNR	SSIM
[^18^F]FDG	NASC	80.28 ± 2.46	76.82 ± 9.57	43.90 ± 6.66	0.9132 ± 0.0163
	CZ-ASC	8.51 ± 7.32	4.77 ± 6.91	56.46 ± 8.17	0.9825 ± 0.0057
	DL-ASC	**7.36 ± 6.77**	**3.66 ± 3.83**	**58.18 ± 6.39**	**0.9828 ± 0.0055**
[^18^F]FAPI	NASC	80.56 ± 2.84	78.68 ± 9.09	45.11 ± 5.74	0.8972 ± 0.0186
	NFT-ASC	12.82 ± 6.14	7.94 ± 5.39	55.43 ± 5.65	0.9632 ± 0.0091
	CZ-ASC	**6.44 ± 7.02**	**2.76 ± 2.11**	**59.68 ± 5.67**	**0.9863 ± 0.0026**
	DL-ASC	7.25 ± 6.33	5.39 ± 5.52	58.66 ± 5.66	0.9732 ± 0.0099
	FT-ASC	6.55 ± 5.89	4.78 ± 4.51	59.18 ± 5.56	0.9756 ± 0.0070
[^68^Ga]FAPI	NASC	80.19 ± 2.52	79.42 ± 5.24	44.30 ± 6.23	0.9289 ± 0.0161
	NFT-ASC	19.51 ± 6.41	9.31 ± 4.03	54.02 ± 7.37	0.9753 ± 0.0056
	CZ-ASC	**5.53 ± 3.99**	**1.92 ± 0.76**	**61.51 ± 7.82**	**0.9859 ± 0.0018**
	DL-ASC	5.68 ± 4.12	3.41 ± 4.38	59.46 ± 7.31	0.9852 ± 0.0027
	FT-ASC	5.60 ± 4.02	2.83 ± 2.51	60.80 ± 6.90	0.9857 ± 0.0021

The best results for each metric are highlighted in bold font

**Fig. 7:**
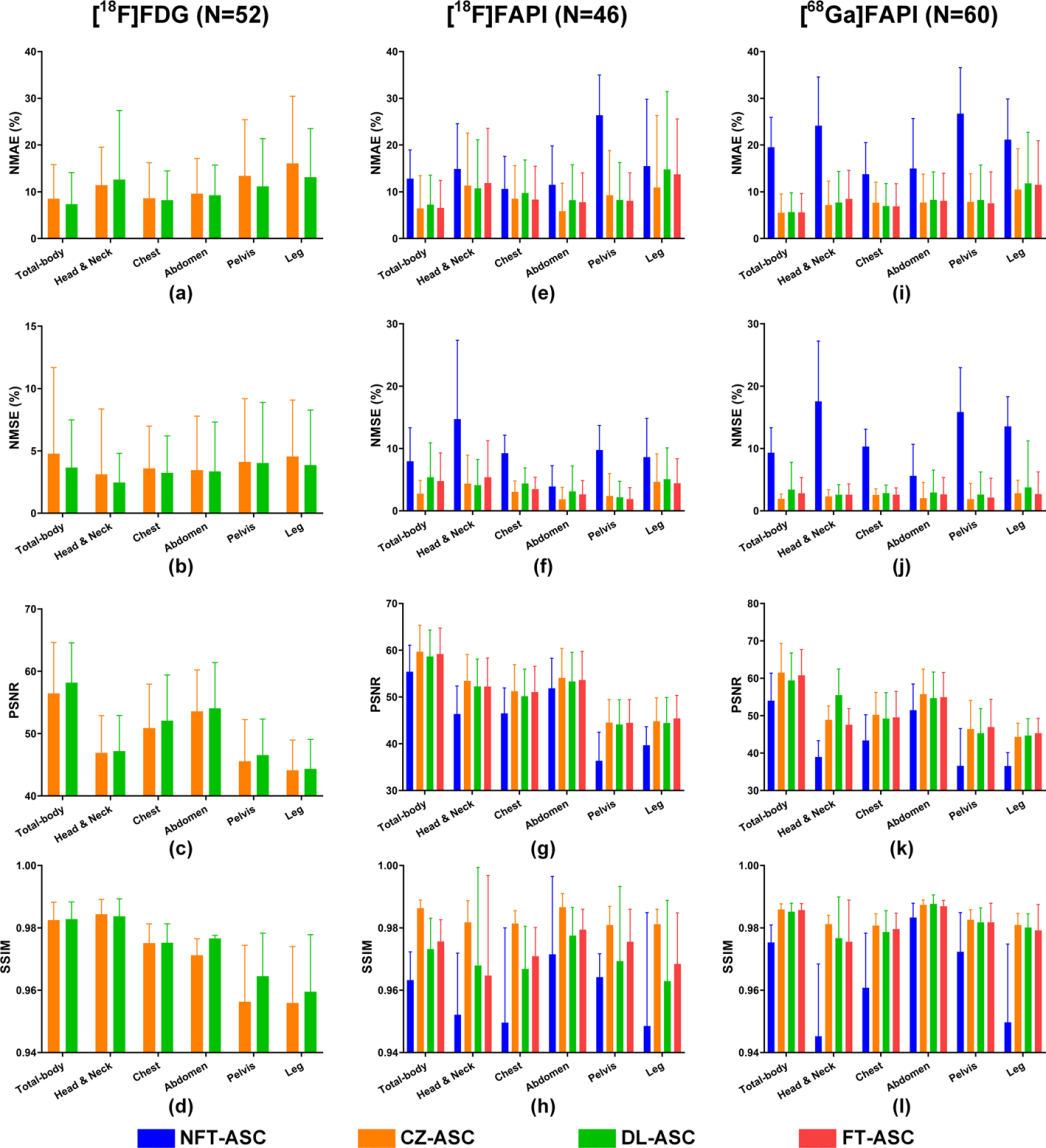
Quantitative error (mean and SD) measured in 5 anatomical regions and total-body PET for different approaches over all subjects in the **a**–**d** [^18^F]FDG, **e**–**h** [^18^F]FAPI, and **i**–**l** [^68^Ga]FAPI datasets in terms of NMAE, NMSE, PSNR and SSIM metrics

Figure 7 depicts the voxel-based quantitative errors (mean and SD) in complete total-body PET and five anatomical regions for different methods over all subjects in three datasets. DL-ASC shows higher PSNR results than CZ-ASC across the head & neck region (DL-ASC: 47.21 ± 5.71, CZ-ASC: 46.92 ± 5.98), chest (DL-ASC: 52.08 ± 7.33, CZ-ASC: 50.89 ± 7.05), abdomen (DL-ASC: 54.08 ± 7.32, CZ-ASC: 53.60 ± 6.63), pelvis (DL-ASC: 46.57 ± 5.77, CZ-ASC: 45.57 ± 6.68), and leg (DL-ASC: 44.35 ± 4.72, CZ-ASC: 44.12 ± 4.85) regions for the [^18^F]FDG dataset. For [^18^F]FAPI and [^68^Ga]FAPI datasets, CZ-ASC generally exhibits the best performance in all anatomical regions, while NFT-ASC shows the poorest performance in each anatomical region. FT-ASC shows higher PSNR results than DL-ASC in the chest ([^18^F]FAPI: FT-ASC: 51.04 ± 5.50, DL-ASC: 50.14 ± 5.82; [^68^Ga]FAPI: FT-ASC: 49.57 ± 6.90, DL-ASC: 49.20 ± 6.98), abdomen ([^18^F]FAPI: FT-ASC: 53.61 ± 6.13, DL-ASC: 53.32 ± 6.22; [^68^Ga]FAPI: FT-ASC: 54.95 ± 6.61, DL-ASC: 54.70 ± 6.98), pelvis ([^18^F]FAPI: FT-ASC: 44.46 ± 4.98, DL-ASC: 44.12 ± 5.28; [^68^Ga]FAPI: FT-ASC: 46.94 ± 7.43, DL-ASC: 45.34 ± 6.54), and leg regions ([^18^F]FAPI: FT-ASC: 45.42 ± 4.92, DL-ASC: 44.81 ± 5.01; [^68^Ga]FAPI: FT-ASC: 45.31 ± 3.99, DL-ASC: 44.70 ± 4.54), but shows inferior PSNR as compared to DL-ASC in the head & neck region ([^18^F]FAPI: FT-ASC: 52.21 ± 6.09, DL-ASC: 52.25 ± 5.85; [^68^Ga]FAPI: FT-ASC: 47.58 ± 4.31, DL-ASC: 55.49 ± 7.00).

**Fig. 8:**
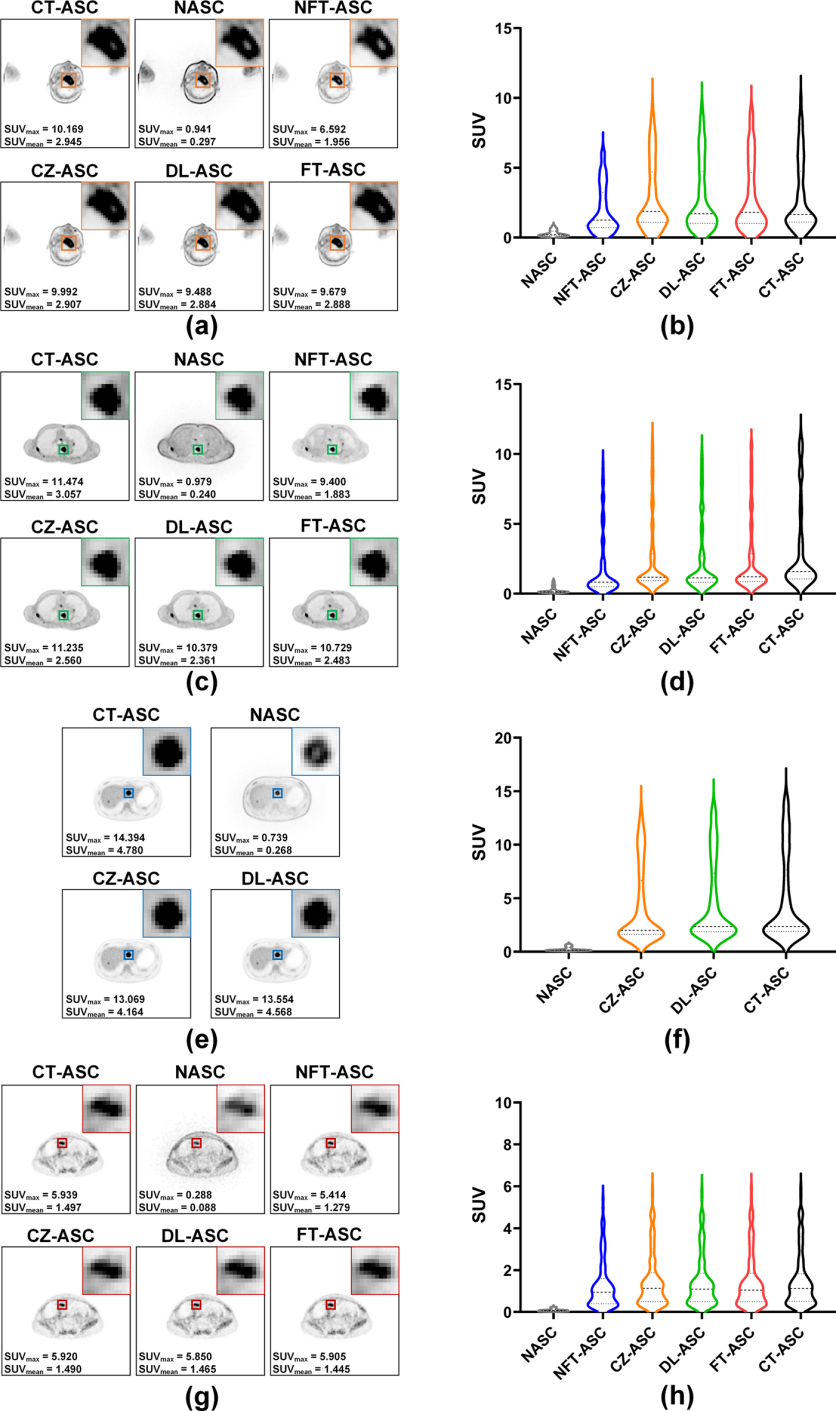
Sample tumor results of CT-ASC, NASC, NFT-ASC, CZ-ASC, DL-ASC and FT-ASC methods in **a**, **b** head-neck region of a male patient (Age: 65, BMI: 23.11, nasopharyngeal carcinoma) in [^18^F]FAPI dataset, **c**, **d** chest region of a female patient (Age: 65, BMI: 23.11, bone tumor) in [^18^F]FAPI dataset, **e**, **f** abdomen region of a male patient (Age: 29, BMI: 22.21, liver tumor) in [^18^F]FDG dataset, and **g**, **h** pelvis region of a female patient (Age: 52, BMI: 21.72, ovarian cancer) in [^68^Ga]FAPI dataset. The enlarged ROIs are displayed in the top right corner of the corresponding image. The SUV_max_ and SUV_mean_ values are shown in the bottom left corner of the corresponding image. The SUV results of ROIs for different methods are presented using violin plots

Figure 8 illustrates four sample tumor results of CT-ASC, NASC, NFT-ASC, CZ-ASC, DL-ASC and FT-ASC methods in different regions. The enlarged ROIs of tumors are displayed in the top right corner of the corresponding image. The SUV results of tumor regions for different methods are presented using violin plots. For the sample tumors in the head & neck, chest, and pelvis regions, CZ-ASC has the closest median, upper, and lower quartile scores to CT-ASC. The SUV results of FT-ASC are the second closest to those of CT-ASC. DL-ASC is slightly worse than FT-ASC overall, except for tumors in the head & neck region. NFT-ASC shows significantly underestimated SUV results yet still outperforms NASC. For the abdominal region tumor, DL-ASC has median, upper, and lower quartile scores closer to CT-ASC compared to FT-ASC. NASC has significantly lower SUV values than CT-ASC in all regions, and the visual tumor volumes in the chest, abdomen, and pelvis regions are notably smaller than CT-ASC.

**Fig. 9:**
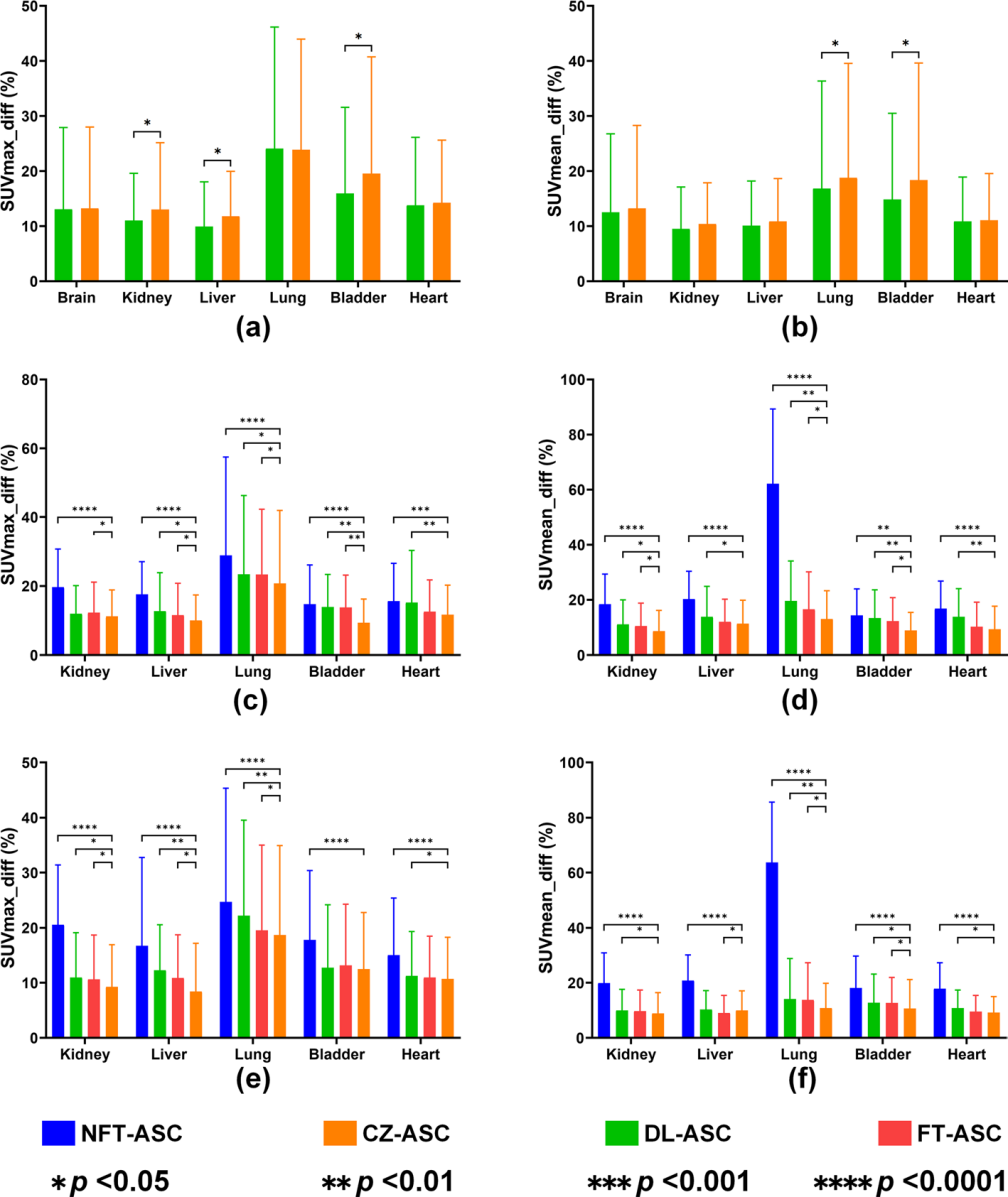
Quantitative error (mean and SD) measured in different organ regions for different approaches across all subjects in **a**, **b** [^18^F]FDG, **c**, **d** [^18^F]FAPI, and **e**, **f** [^68^Ga]FAPI datasets in terms of SUV_max__diff and SUV_mean__diff. A paired *t*-test with Bonferroni correction was used to measure the results in target regions between CZ-ASC and other methods. A *p*-value < 0.05 indicates a significant difference

Figure 9 shows SUV_max__diff and SUV_mean__diff results in different regions for the different methods over all subjects in the three datasets. The highest SUV_max__diff and SUV_mean__diff appeared in the lung region for all methods in the three datasets. For [^18^F]FDG dataset, DL-ASC shows a smaller SUV_max__diff than CZ-ASC in the kidney, liver and bladder regions with significant difference (*p* < 0.05). It also shows a significantly smaller SUV_mean__diff than CZ-ASC in lung and bladder regions (*p* < 0.05). DL-ASC has a larger SUV_max__diff (DL-ASC: 24.10 ± 22.05%, CZ-ASC: 23.87 ± 20.08%) than CZ-ASC in the lung region but without significance (*p* > 0.05). For [^18^F]FAPI and [^68^Ga]FAPI datasets, CZ-ASC performs the best with the smallest SUV_max__diff and SUV_mean__diff in each organ region, while NFT-ASC has the largest SUV_max__diff and SUV_mean__diff in each organ region. DL-ASC is worse than CZ-ASC for SUV_max__diff and SUV_mean__diff results in almost all regions with significant differences (*p* < 0.05) in the [^18^F]FAPI dataset. FT-ASC is significantly worse (*p* < 0.05) than CZ-ASC for SUV_max__diff results in the kidney, liver, lung and bladder regions and SUV_mean__diff results in the kidney, lung and bladder regions in the [^18^F]FAPI dataset. In the [^68^Ga]FAPI dataset, DL-ASC shows worse performance than CZ-ASC for SUV_max__diff results in the kidney, liver, lung and heart regions with significant differences (*p* < 0.05). It also shows worse performance in terms of SUV_mean__diff in the kidney, bladder, and heart regions with a significant difference (*p* < 0.05). FT-ASC is significantly worse than CZ-ASC (*p* < 0.05) for SUV_max__diff in the kidney, liver and lung regions and SUV_mean__diff in the liver and bladder regions.

**Fig. 10:**
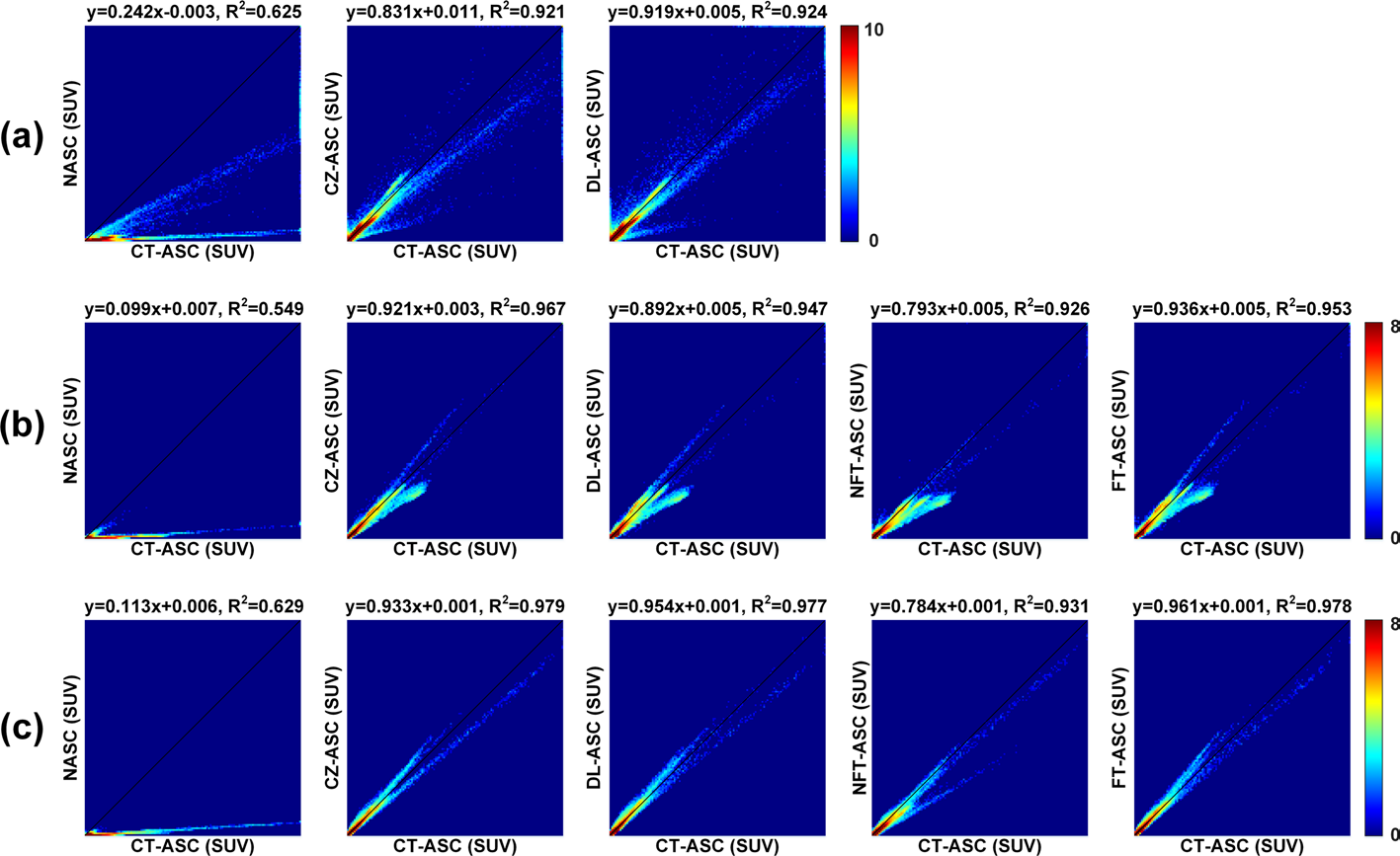
Joint histogram and linear regression analysis of different methods on total-body PET images for all subjects in **a** [^18^F]FDG (*N* = 52), **b** [^18^F]FAPI (*N* = 46) and **c** [^68^Ga]FAPI (*N* = 60) dataset, respectively. The CT-ASC is used as reference

Figure 10 illustrates the joint histogram and linear regression analysis results of different AC methods on total-body PET images across all tested subjects in three datasets using CT-ASC as the reference. CZ-ASC (slope = 0.831, *R*^2^ = 0.921) and DL-ASC (slope = 0.919, *R*^2^ = 0.924) show higher correlations with CT-ASC than NASC (slope = 0.242, *R*^2^ = 0.625) in [^18^F]FDG dataset. For [^18^F]FAPI and [^68^Ga]FAPI datasets, CZ-ASC ([^18^F]FAPI: slope = 0.921, *R*^2^ = 0.967; [^68^Ga]FAPI: slope = 0.933, *R*^2^ = 0.979) shows the highest correlations with the reference CT-ASC. DL-ASC ([^18^F]FAPI: slope = 0.892, *R*^2^ = 0.947; [^68^Ga]FAPI: slope = 0.954, *R*^2^ = 0.977) and FT-ASC ([^18^F]FAPI: slope = 0.936, *R*^2^ = 0.953; [^68^Ga]FAPI: slope = 0.961, *R*^2^ = 0.978) are better than NASC ([^18^F]FAPI: slope = 0.099, *R*^2^ = 0.549; [^68^Ga]FAPI: slope = 0.113, *R*^2^ = 0.629) in both [^18^F]FAPI dataset [^68^Ga]FAPI dataset. We can also observe that FT-ASC shows higher correlations with CT-ASC than DL-ASC. NFT-ASC ([^18^F]FAPI: slope = 0.793, *R*^2^ = 0.926; [^68^Ga]FAPI: slope = 0.784, *R*^2^ = 0.931) is worse than DL-ASC and FT-ASC, yet better than NSAC.

## Discussion

This work demonstrates the feasibility of directly generating attenuation and scatter-corrected images from NASC images for multi-tracer total-body PET using different AI-based ASC strategies, including DL-ASC, CZ-ASC, NFT-ASC and FT-ASC. Qualitative and quantitative results show that DL-ASC, CZ-ASC and FT-ASC are feasible in producing tracer distribution estimations that correlate closely with the reference CT-ASC. CZ-ASC and FT-ASC can outperform DL-ASC in terms of various qualitative and quantitative results for cross-tracer total-body PET.

DL-ASC approaches usually require large, reliable datasets to generate robust and generalizable models [55]. Though DL-ASC may achieve reliable performance in the internal validation dataset, it may not yield favorable results when tested on cross-tracer PET datasets due to significant dataset variations. In our study, we test all subjects from [^18^F]FAPI and [^68^Ga]FAPI datasets on the pre-trained cross-tracer [^18^F]FDG network directly, i.e., NFT-ASC. Various results show that NFT-ASC can improve tumor uptake contrast as compared to NASC, but it still produces significant errors with the reference CT-ASC. This issue could be attributed to significant variations in tracer distribution and image features among different tracers, as well as the insufficient amount of training data available. These factors present challenges in accurately generating anatomical structures and tracer distribution for total-body PET.

CZ-ASC and FT-ASC could exhibit superior performance as compared to DL-ASC, showing great potential for cross-tracer total-body PET. In our study, CZ-ASC achieved the best performance in [^18^F]FAPI and [^68^Ga]FAPI datasets, but it was worse than DL-ASC in [^18^F]FDG dataset. This discrepancy could be attributed to the data imbalance in the centralized server, with a twofold difference in data quantity between FAPI and FDG. CZ-ASC was implemented by directly mixing different data types into one server to train a robust network model. Compared with DL-ASC and FT-ASC, CZ-ASC increased the sample size of training data and had the potential to achieve the best performance. However, due to the imbalance in the quantity of FAPI and FDG data, CZ-ASC might prefer to capture FAPI tracer features, potentially losing essential patterns in the FDG data. On the other hand, FT-ASC re-used a pre-trained DL-ASC model from other existing data instead of starting the training from scratch, which is equivalent to increasing the sample size of the training data compared to DL-ASC. Therefore, it is obvious that FT-ASC outperforms DL-ASC. FT-ASC performed better than DL-ASC in the chest, abdomen, pelvis and leg regions but not head & neck region. In contrast to [^18^F]FDG, [^18^F]FAPI and [^68^Ga]FAPI have low physiological uptake in the brain for the subjects without brain metastases [56]. Though the pre-trained [^18^F]FDG network model was fine-tuned by [^18^F]FAPI or [^68^Ga]FAPI data, it still retains a substantial number of data pattern characteristics from [^18^F]FDG. Consequently, this leads to subpar performance of FT-ASC in the head & neck region for [^18^F]FAPI and [^68^Ga]FAPI datasets. Additionally, previous studies [48] have demonstrated that promising results could be achieved by FT pre-trained networks with only a small amount of data, but the evaluation of these strategies is beyond the scope of this study.

CZ-ASC, DL-ASC, and FT-ASC have promising potential to eliminate the possibility of CT misregistration, reduce CT radiation dose and subsequent patient cancer risk, and omit the additional reconstruction step with time-consuming computation for total-body imaging. From a technical perspective, these three methods can eliminate the need for attenuation map generation, which is an essential step in conventional PET image reconstruction. The subsequent additional PET reconstruction step tends to be time-consuming, especially for total-body PET, which takes longer than traditional PET/CT. DL-ASC, CZ-ASC, and FT-ASC provide a simpler and faster alternative (< 1 s) as compared to the conventional reconstruction step, improving clinical examination efficiency. For clinical applications, CT and PET data mismatches are common in total-body PET/CT imaging, such as in the liver dome region. This is due to involuntary motion (respiratory, heart and diaphragm) and voluntary motion (patient movement) between the sequential CT and PET scans. DL-ASC, CZ-ASC, and FT-ASC have promising potential to eliminate the possibility of CT misregistration, which would be beneficial to doctors in accurately detecting lesion areas and patients who struggle with breath-holding. DL-ASC, CZ-ASC, and FT-ASC could also eliminate the need for multiple CT scans, significantly reducing the radiation associated with CT scans. This advancement would benefit patients requiring multiple examinations, especially pregnant and pediatric patients. The data used in this study consist of 5-min total-body PET scans. Compared to standard 20-min acquisition times, the 5-min total-body PET images have slightly higher noise but still maintain diagnostic quality [57]. DL-ASC, CZ-ASC, and FT-ASC methods could be considered valid for lower-dose total-body PET AC.

Although our methods have demonstrated feasibility across various organs and anatomical regions, it is essential to note the presence of significant SUV errors in certain regions. Specifically, DL-ASC, FT-ASC, and CZ-ASC have the largest SUV errors in the lung region, which could be attributed to respiratory motion during image acquisition. These motions lead to considerable variations in tracer uptake measurements and a lack of consistency between adjacent slices. This issue has also been reported by Izadi et al. [54]. Furthermore, we found that quantification errors in SUV_max_ are greater than those in SUV_mean_ for DL-ASC, FT-ASC, and CZ-ASC. This suggests a risk of influence from outliers for DL-ASC, FT-ASC, and CZ-ASC. This risk is related to end-to-end network training for direct mapping. Compared to standard 20-min total-body PET scanning, the data used in this study still contain a certain degree of noise, which directly affects the performance of end-to-end mapping for DL-ASC, FT-ASC, and CZ-ASC. Previous studies have shown that DL-based estimation of attenuation map for AC can outperform direct generation of AC SPECT [58, 59]. This strategy could be adopted for total-body PET ASC to address the limitations of end-to-end direct mapping. Furthermore, the large regional biases observed in the error maps, particularly in the brain region as shown in Figs. 3 and 5, may limit the clinical applicability of the proposed methods for that specific region. In the future, we plan to further optimize our approach to be organ-aware, which will enhance its adaptability to different organ regions.

There are some limitations in this study. Firstly, the clinical application of CZ-ASC may pose challenges due to the need for data pooling to a single server, which raises privacy concerns about patient data and is limited by imbalanced data sample sizes in different datasets. Additionally, FT-ASC relies on sufficient pre-training data, and inadequately pre-trained networks may propagate errors to the fine-tuned model, resulting in image artifacts. Transfer learning based on simulation data may be a practical solution to this issue [60]. Furthermore, the error maps may exhibit activity discontinuities, particularly between the pelvis and thighs, although these discontinuities are less pronounced in the SUV images. The use of image patches likely causes this discrepancy. The significant difference in bone density between the pelvis and thighs can lead to discrepancies in the network’s output images, especially at the image edges, making image stitching a challenging task. The datasets used in this work are still relatively small, though the data augmentation technique is implemented. Another limitation of the datasets is the lack of ultra-low-dose and pediatric PET data. Additionally, the datasets do not include instances of image artifacts. Therefore, further validation is warranted to evaluate the model’s generalizability on a broader range of cases. Due to the scarcity of clinical total-body PET data and the privacy concerns associated with clinical data, we have not yet tested our network models on external datasets. Therefore, further evaluation with larger patient cohorts from different centers is warranted. The network used in this study is a fully convolutional architecture, with an underlying translation-equivariance property. This property allows the network to adapt to various patch sizes as inputs, which can reduce stitching artifacts caused by overlapping small image patches [42]. Further investigation is warranted to evaluate the training of network models using variable patch sizes as inputs. Although the 3D cGAN used in this study shows effective performance, it may not be the most suitable network architecture for direct ASC in multi-tracer total-body PET imaging. Another study has explored the application of a cycle-consistent generative adversarial network (Cycle-GAN) for low-dose total-body PET AC [61]. Further exploration is warranted to investigate more effective network architectures. Lastly, a systematic clinical evaluation comparing different AI-based ASC methods is warranted for further investigation.

## Conclusions

This work demonstrated the feasibility of directly generating attenuation and scatter-corrected images from NASC images based on a 3D cGAN framework for multi-tracer total-body PET. We further compared the performance of different AI-based ASC strategies using various qualitative and quantitative evaluations. Our experimental results showed that DL-ASC, CZ-ASC and FT-ASC had great potential to produce highly correlated tracer distribution estimations and achieve comparable performances with clinical CT-ASC. CZ-ASC and FT-ASC could outperform DL-ASC and have great potential for cross-tracer total-body PET. Overall, DL-ASC, CZ-ASC and FT-ASC are promising for routine total-PET clinical practice.

## Data Availability

The datasets used and/or analysed during the current study are available from the corresponding author on reasonable request.

## Abbreviations

[^18^F]FDG: [^18^F]-fluorodeoxyglucose
[^18^F]FAPI: [^18^F]-fibroblast-activation protein inhibitors
[^68^Ga]FAPI: [^68^Ga]-fibroblast-activation protein inhibitors
SUV: Standardized uptake value
ASC: Attenuation and scatter correction
LAFOV: Long axial field of view
AC: Attenuation correction
CT-ASC: CT-based ASC
MLAA: Maximum-likelihood reconstruction of attenuation and activity
µ-maps: Attenuation maps
TOF: Time-of-flight
AI: Artificial intelligence
DL: Deep learning
NAC: Non-attenuation-corrected
FT: Fine-tuning
CNN: Convolutional neural network
ACFs: Attenuation correction factors
MP: Myocardial perfusion
cGAN: Conditional generative adversarial network
NASC: Non-attenuation and scatter-corrected
OSEM: Ordered subset expectation maximization
PSF: Point-spread function
IN: Instance normalization
ReLU: Rectified linear unit
LReLU: Leaky rectified linear unit
BN: Batch normalization
NMAE: Normalized mean absolute error
NMSE: Normalized mean square error
PSNR: Peak signal-to-noise ratio
SSIM: Structural similarity index
ROI: Regions of interest
